# Prevalence of selected bleeding and thrombotic events in persons with hemophilia versus the general population: A scoping review

**DOI:** 10.1016/j.rpth.2022.100007

**Published:** 2022-12-09

**Authors:** Amy D. Shapiro, Brandon M. Hardesty, Flora Peyvandi, Alfonso Iorio

**Affiliations:** 1 Indiana Haemophilia and Thrombosis Center, Indianapolis, Indiana, USA; 2 Fondazione Istituto di Ricovero e Cura a Carattere Scientifico Ca’ Granda Ospedale Maggiore Policlinico Angelo Bianchi Bonomi Hemophilia and Thrombosis Center, Milan, Italy; 3 Department of Pathophysiology and Transplantation, Università degli Studi di Milano, Milan, Italy; 4 Department of Health Research Methods, Evidence, and Impact, McMaster University, Hamilton, Ontario, Canada; 5 Department of Medicine, McMaster University, Hamilton, Ontario, Canada

**Keywords:** cardiovascular diseases, hemophilia A, hemophilia B, hemorrhage, mortality, thrombosis

## Abstract

Life expectancy for persons with hemophilia has increased over recent decades due to advances in treatment practice and patient care. Those with hemophilia are now more likely to be affected by conditions associated with aging, such as myocardial infarction, hemorrhagic/ischemic stroke, deep vein thrombosis, pulmonary embolism, and intracranial hemorrhage. Here, we describe the results of a literature search designed to summarize current data on the prevalence of the above selected bleeding and thrombotic events in persons with hemophilia vs the general population. A total of 912 articles published between 2005 and 2022 were identified in a search of BIOSIS Previews, Embase, and MEDLINE databases conducted in July 2022. Case studies, conference abstracts, review articles, studies focusing on hemophilia treatments or surgical outcomes, and studies examining patients with inhibitors only were excluded. After screening, 83 relevant publications were identified. The prevalence of bleeding events was consistently higher in hemophilia populations vs reference populations (hemorrhagic stroke, 1.4%-5.31% vs 0.2%-0.97%; intracranial hemorrhage, 1.1%-10.8% vs 0.04%-0.4%). Serious bleeding events showed a high rate of mortality with standardized mortality ratios for intracranial hemorrhage ranging from 3.5 to 14.88. Although 9 studies reported lower prevalence of arterial thrombosis (myocardial infarction/stroke) in hemophilia vs general populations, 5 studies reported higher or comparable prevalence in hemophilia. Prospective studies are therefore needed to understand the prevalence of bleeding and thrombotic events in hemophilia populations, particularly with the observed increases in life expectancy and availability of novel treatments.

## Essentials

• We compare the prevalence of bleeding/thrombotic events in hemophilia vs general population.
• Bleeding event prevalence was consistently higher in persons with hemophilia than those without hemophilia.
• Data suggest that the prevalence of thrombotic events is not always lower in populations with hemophilia.
• Prospective cohort studies are needed to examine bleeding/thrombosis prevalence in hemophilia.

## 1 Introduction

Hemophilia A and hemophilia B are rare bleeding disorders caused by congenital deficiencies in coagulation factors VIII or IX, respectively. Advances in treatment and management over recent decades have led to vast improvements in the quality of life for persons with hemophilia [1]. Prophylactic treatment with recombinant or plasma-derived factor concentrates, the current standard of care for many patients, has been advanced by the availability of extended half-life factor products that require less frequent intravenous dosing. Nonfactor therapies such as emicizumab, concizumab, marstacimab, and fitusiran are either available or are under clinical trials as novel therapies for patients with and without inhibitors, with the major advantage of subcutaneous administration.

Life expectancy for persons with hemophilia has increased as a result of these advancements, and an increasing number of patients are now more likely to be affected by conditions typically associated with an aging population [2]. This includes a range of cardiovascular (CV) complications such as myocardial infarction, hemorrhagic/ischemic stroke, deep vein thrombosis, pulmonary embolism, and intracranial hemorrhage (ICH). However, currently, there are gaps in our knowledge regarding the prevalence of these events in hemophilia populations. For example, it is unclear whether the prevalence of these events is comparable with that of the general population and whether persons with hemophilia are protected from thrombosis due to the nature of their condition despite the advances in replacement therapy regimens. Historically, persons with hemophilia have been thought to be less likely affected by thrombotic conditions due to hypocoagulability, which is associated with reduced thrombin generation [3]. However, the literature appears to present conflicting evidence regarding the prevalence of CV events in hemophilia populations vs the general population. Although many studies support the idea that prevalence of CV events is generally higher in the general population [4,5], conversely, several studies have found that, at least in some populations, prevalence of certain thrombotic CV conditions is in fact higher in those with hemophilia [[6], [7], [8]]. There is therefore uncertainty regarding the protective effect of hemophilia under thrombotic conditions.

In addition to the studies investigating the prevalence of CV events, studies have also evaluated the mortality associated with these conditions in persons with hemophilia compared with that in the general population [2,9,10]. Although ICH is one of the leading causes of death in persons with hemophilia, mortality due ischemic heart disease has been reported to be lower in persons with hemophilia [9]. The question remains as to whether all-cause or cause-specific mortality is decreasing over time for persons with hemophilia and whether this can be concluded based on the currently available data.

A scoping review was designed to extract and summarize currently available data on the prevalence of the selected bleeding and thrombotic events and mortality in populations of persons with hemophilia. Special attention was given specifically to publications that compared the prevalence in hemophilia with that in matched controls of the general population. Scoping reviews differ from systematic reviews in that they do not aim to answer a specific research question or understand the mechanisms that explain the identified data [11]. This approach is used when it is unclear whether more specific research questions can be answered with the currently available data (eg, whether persons with hemophilia are protected from thrombosis). The aim of this review was therefore to provide a comprehensive overview of the current state of the literature in these areas (bleeding, thrombosis, and mortality) to identify and discuss relevant research gaps that could be addressed in the future.

## 2 Methods

### 2.1 Search strategy

A search strategy was developed to identify publications reporting data on the prevalence of selected bleeding and thrombotic events and mortality in persons with hemophilia (Supplementary Table 1). Search terms (Medical Subject Headings) were chosen to include the bleeding (ICH, gastrointestinal bleeding, and hemorrhagic stroke) and thrombotic (deep vein thrombosis, myocardial infarction, thrombotic stroke, thrombotic microangiopathy, and pulmonary embolism) events selected for analysis. As hemorrhagic stroke can be reported as both stroke with hemorrhagic transformation and ICH, the search terms were selected for both events to capture all relevant publications. Terms were also included to identify publications with data on mortality. Studies focusing on persons with hemophilia A and B were incorporated. The chosen publications were in English and published between January 1, 2005, and July 19, 2022. This strategy shown in Supplementary Table 1 was first used to search the following databases on January 2, 2021: BIOSIS Previews, Embase, and MEDLINE. The search was also repeated on July 19, 2022. A protocol was not created for this review article; the Preferred Reporting Items for Systematic Reviews and Meta-Analyses extension for scoping reviews checklist [12] was used to guide drafting of the manuscript.

### 2.2 Screening

A flowchart of the process used to screen the abstracts identified in the search is provided in the Figure. To identify peer-reviewed studies reporting primary data on the prevalence of the selected events in the populations of persons with hemophilia, the following publication types were excluded: case studies, conference abstracts, posters, guidelines, consensus statements, editorials, comments, and reviews. The following study types were also excluded as they were deemed highly unlikely to contain relevant data on the prevalence of CV conditions in persons with hemophilia: studies not directly related to hemophilia, studies focusing on cancer (eg, those comparing prevalence of cancer types in hemophilia), preclinical/animal studies, studies investigating persons with acquired hemophilia, studies investigating specific hemophilia treatments (eg, safety, efficacy, and pharmacokinetic and pharmacodynamic studies), cost-effectiveness studies, studies examining patients with inhibitors only (studies including both inhibitor and noninhibitor patients were considered), and studies with a focus on surgical outcomes. Studies investigating specific hemophilia treatments were excluded as they had narrow inclusion criteria and reported pharmacokinetic/ pharmacodynamic/efficacy/safety data instead of comparing the event prevalence in large populations of persons with hemophilia with that in the general population. Studies on surgical outcomes were excluded due to confounding issues, such as the wide range of procedures and patients included and the use of different replacement and thromboprophylaxis regimens. Abstracts not excluded based on these criteria were assessed based on the likelihood that they could potentially contain data on the prevalence of the selected events in persons with hemophilia. Those abstracts with a clear indication that no relevant data were to be found were assigned to a “not relevant” category. In cases where there was an indication that relevant data could potentially be included (either specifically mentioned or alluded to in the text), full-text articles were reviewed for data extraction.

**Figure:**
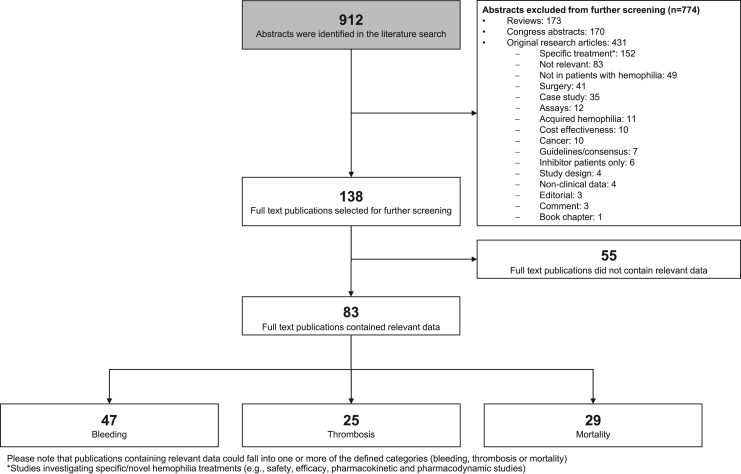
Screening flow chart.

### 2.3 Data extraction

Full-text publications of selected abstracts were screened to identify data on the prevalence of the selected adverse events in persons with hemophilia. For each full-text publication, review of the results section and any accompanying figures or tables was performed. For those containing relevant data, this information was then extracted and documented in 3 separate spreadsheets (bleeding, thrombosis, or mortality). Within each spreadsheet, data were sorted into 2 tabs: 1) Publications comparing prevalence/mortality between hemophilia and general populations and 2) publications reporting prevalence/mortality in hemophilia populations only. Some publications were included in more than one spreadsheet. Those studies not containing any relevant prevalence or mortality data were assigned again to the “not relevant” category.

## 3 Results

### 3.1 Search results

A total of 912 references were identified in the literature search, 774 of which were excluded from further screening based on the exclusion criteria (Figure). Full-text publications for the remaining abstracts (n = 138) were then screened to extract relevant data on the prevalence of the selected events in persons with hemophilia. A further 55 full-text publications were excluded based on the screening criteria, leaving 83 relevant publications that were divided into 3 categories: bleeding, thrombosis, and mortality.

### 3.2 Bleeding

There were 47 publications reporting data on the prevalence of the selected bleeding events (hemorrhagic stroke, ICH, and gastrointestinal bleeding) in persons with hemophilia. Of these, 10 publications included comparisons with the general population (Table 1) [[4], [5], [6], [7],[13], [14], [15], [16], [17], [18]]. Nine of these 10 studies compared the prevalence in persons with hemophilia with matched controls [[4], [5], [6], [7],[13], [14], [15],^19^]. The prevalence of the selected events was observed to be consistently higher in hemophilia populations compared with that in the general population. The prevalence of hemorrhagic stroke was reported to be higher for those with hemophilia in 4 studies (1.4%-5.31% in hemophilia vs 0.2%-0.97% in the general population). Overall, the prevalence of ICH was reported in 4 publications and ranged from 1.1% to 10.8% in hemophilia vs 0.04% to 0.4% in the general population. When evaluating ICH data by age, prevalence was reported for newborns with hemophilia in 2 of the 4 studies (3.4% and 3.1%), which was significantly higher than that in the general population (0.04%-0.11%) [14,16]. In the remaining 2 studies, the overall prevalence of nonfatal ICH is reported for adults with hemophilia as 1.6% (≥30 years) [4] and 7.4% (≥40 years) [5], with 0.4% being affected in the general population in both studies. The prevalence of ICH was lower in nonsevere (1.1% and 3.9%) vs severe adults with hemophilia (2.0% and 10.8%), respectively. One study assessed the risk of upper gastrointestinal bleeding associated with *Helicobacter pylori* infection (31.5% in hemophilia vs 2.0% in the general population) [13].

**Table 1:** Prevalence of selected bleeding events in persons with hemophilia vs the general population.

References	Patients and age	Years studied	Bleeding event	Hemophilia population			General population		
Szczepanik et al. 2005 [13]	HA/B 17-63 y	2000-2002	Upper gastrointestinal bleeding *H. pylori* positive *H. pylori* negative Total	No. of persons with HA/B (%) 72 (49.3) 74 (50.7) 146 (100.0)	Episodes of bleeding in history (%) 33 (71.7) 13 (28.3) 46 (31.5)	No episodes of bleeding in history (%) 39 (39.0) 61 (61.0) 100 (68.5)	No. of age-matched patients (%) 39 (39.0) 61 (61.0) 100 (100.0)	Episodes of bleeding in history (%) 2 (2.0) - 2 (2.0)	No episodes of bleeding in history (%) 37 (37.8) 61 (62.2) 98 (98.0)
Tarantino et al. 2007 [14]	Newborns with hemophilia	1988-2001	Intracranial hemorrhage	Newborns with hemophilia n = 580 20 (3.4%)			Newborns without hemophilia n = NR 0.11%; *P* < .0001		
Fransen van de Putte et al. 2012 [4]	HA/B ≥30 y	2009-2011	Intracranial bleeding	HA/B overall N = 709 1.6% (0.8-2.8)	Severe n = 344 2.0% (0.8-4.2)	Nonsevere n = 365 1.1% (0.3-2.8)	Age-matched general male population n = NR 0.4% (0.2-0.6)		
Fransen van de Putte et al. 2012 [5]	HA/B ≥40 y	1985-2010	Intracranial bleeding	HA/B overall N = 408 7.4% (5.0-10.3)	Severe n = 204 10.8% (6.9-15.9)	Nonsevere n = 204 3.9% (1.7-7.6)	Age-matched general male population n = NR 0.4% (0.35-0.43)		
Pocoski et al. 2014 [7]	HA All ages	2007-2009	Hemorrhagic stroke Hemorrhagic stroke (excluding HIV and/or hepatitis C)	Persons with HA n = 2506 2.0% n = 1995 39 (2.0%)			Persons without HA (matched 1:3) n = 7518 0.5%; *P* < .001 n = 5985 30 (0.5%); *P* < .001		
Wang et al. 2015 [15]	HA/B All ages	1997-2010	Hemorrhagic stroke	Persons with HA/B n = 1054 56 (5.31%)			Age/sex-matched general population n = 10,540 78 (0.74%) Odds ratio: 7.53 (95% CI, 5.31-10.67); *P* < .05		
Humphries et al. 2016 [6]	HA All ages	2008-2011	Hemorrhagic stroke	Persons with HA n = 1050 1.4%			Persons without HA (matched 1:3) n = 3150 0.2%; *P* < .0001		
Nazir et al. 2016 [16]	Newborns <2 wks old with HA	1998-2015	Intracranial hemorrhage	Newborns with HA n = 163 5 (3.1%)			Nonhemophilic children n = NR 0.04-0.11% (estimated)^a^		
Chu et al. 2018 [17]	HA/B >16 y	1997-2010	Hemorrhagic stroke n, %	Employed persons with hemophilia n = 400 17 (4.29%)			Matched persons in the general population n = 1600 15 (0.97%) Crude HR (95% CI): 4.72 (2.94-7.59); *P* < .0001 Adjusted HR (95% CI): 4.60 (2.81-7.53); *P* < .0001		
Husseinzadeh et al. 2018 [18]	HA/B ≥18 y	2013-2016	Cerebral microbleeds	Persons with HA n = 31 11 (35%; 95% CI, 19%-58%)			Controls n = 32 8 (25%; 95% CI, 11%-47%); *P* = .42		

HA/B, hemophilia A/B; HR, hazard ratio; NR, not reported.

a Estimated from previously published data.

### 3.3 Thrombosis

Prevalence of thrombotic events in hemophilia populations was reported in 25 publications (myocardial infarction, ischemic stroke, arterial and/or venous thrombosis). Of these, 14 publications made comparisons with matched controls from the general population (Table 2) [[4], [5], [6], [7], [8],^15^,[20], [21], [22], [23], [24], [25], [26], [27]]. Most publications reported that the prevalence of thrombotic events was lower in the hemophilia population when compared with that in the general population. However, 3 publications reported higher prevalence of thrombotic events in persons with hemophilia, including myocardial infarction, arterial and venous thrombosis, and ischemic stroke [[6], [7], [8]]. Lövdahl et al. [26] reported comparable prevalence of myocardial ischemia in hemophilia and general populations (8.2% and 8.1%, respectively). Similarly, Faghmous et al. [27] concluded that the prevalence of myocardial infarction and pulmonary embolism was comparable between both populations, and there was a slightly higher prevalence of deep vein thrombosis and ischemic stroke in persons with hemophilia. There was considerable variation in reported prevalence of the selected events between studies. In the 2 studies that split persons with hemophilia by severity [4,5], the prevalence of myocardial infarction and ischemic stroke was lower in patients with severe hemophilia than in persons with nonsevere hemophilia. As expected, studies assessing older patients showed higher prevalence of cardiovascular disease (CVD) than those that assessed patients of all ages [20,21,23,24,27]. When comparing the prevalence of arterial and venous thrombotic events in persons with hemophilia, most studies appear to only report arterial thrombotic events (eg, myocardial infarction and ischemic stroke). Four of 14 studies combined the prevalence of venous or arterial thrombosis into general “venous thrombosis” or “arterial thrombosis” categories [6,7,15,25]. When comparing these studies, there were differences in the prevalence of arterial vs venous events between populations. For arterial thrombosis, prevalence ranges from 0% to 12.1% in hemophilia vs 0.5% to 5.9% in the general population. For venous thrombosis, prevalence ranges from 0.19% to 4.9% in hemophilia vs 0.09% to 5.9% in the general population

**Table 2:** Prevalence of selected thrombotic events in persons with hemophilia vs the general population.

Reference	Patients and age	Years studied	Thrombotic event	Hemophilia population			General population		Higher in general population?
Kulkarni et al. 2005 [20]	HA/B 45-64 and 65+ y	1993-1998	n; rate^a^ Ischemic heart disease	Hospital discharges: males with hemophilia (1993-1998) Age 45-64: n = 50; rate = 24.1 Age 65+: n = 84; rate = 127.3			Hospital discharges: US males (2000) Age 45-64: n = 1,445,727; rate = 48.9 Age 65+: n = 2,527,397; rate = 175.6		Yes
Miesbach et al. 2009 [21]	HA ≥60 y	2006-2008	n (%) Coronary heart disease Myocardial infarction	Elderly persons with HA n = 29 5 (17%) 1 (3%)			Patients with ischemic heart disease^b^ n = NR Ages 60-79: 27% Age ≥80: 34%		Yes
Sharathkumar et al. 2011 [8]	HA/B ≥35 y	2004-2008	Lifetime CVD prevalence Coronary artery disease Stroke Myocardial infarction	Adults with HA/B n = 185 36 (19.5%) Expected cases in study cohort 13.69 5.735 12.21	Observed cases in study cohort 24 13 22	SPR (CI)^c^ 1.75 (1.05–2.45) 2.27 (1.03–3.50) 1.80 (1.05-2.55)	NHANES prevalence rate for males aged ≥18 y^c^ 7.4 3.1 6.6		No
Fransen van de Putte et al. 2012 [4]	HA/B ≥30 y	2009-2011	% (95% CI) Myocardial infarction Stroke overall Ischemic stroke	HA/B overall n = 709 2.7% (1.6-4.2) 2.1% (1.2-3.5) 0.6% (0.2-1.4)	Severe n = 344 1.7% (0.6-3.8) 2.0% (0.8-4.2) 0.0% (0.0-0.9)	Nonsevere n = 365 3.6% (1.2-6.0) 2.2% (1.0-4.3) 1.1% (0.3-2.8)	Age-matched general male population n = NR 4.0% (3.5-4.6) 1.9% (1.6-2.4) 1.5% (1.2-1.9)		Yes Except stroke overall
Fransen van de Putte et al. 2012 [5]	HA/B ≥40 y	1985-2010	% (95% CI) Myocardial infarction Ischemic stroke	HA/B overall n = 408 2.5% (1.2-4.5) 1.0% (0.3-2.5)	Severe n = 204 0.5% (0.0-2.7) 0.5% (0.0-2.7)	Nonsevere n = 204 4.4% (2.0-8.2) 1.5% (0.3-4.2)	Age-matched general male population n = NR 4.8% (4.6-4.9) 1.4% (1.28-1.42)		Yes Except ischemic stroke (nonsevere)
Pocoski et al. 2014 [7]	HA All ages	2007-2009	% Ischemic stroke Coronary artery disease Myocardial infarction Arterial thrombosis Venous thrombosis	Persons with HA n = 2506 4.7% 10.7% 0.8% 12.1% 4.4%			Persons without HA (matched 1:3) n = 7518 2.7%; *P* < .001 5.8%; *P* < .001 0.3%; *P* = .003 5.9%; *P* < .001 1.1%; *P* < .001		No
Wang et al. 2015 [15]	HA/B All ages	1997-2010	% Atherothrombosis Ischemic stroke Coronary artery disease Myocardial infarction Venous thrombosis Pulmonary embolism	Persons with HA/B n = 1054 4.93% 2.47% 2.75% 0.19% 0.19% 0.00%			Age/sex-matched general population n = 10,540 5.72% 2.61% 3.97% 0.45% 0.09% 0.01%		Yes Except venous thrombosis
Humphries et al. 2016 [6]	HA All ages	2008-2011	% Ischemic stroke Coronary artery disease Myocardial infarction Arterial thrombosis Venous thrombosis	Persons with HA n = 1050 4.1% 8.9% 1.4% 9.6% 4.9%			Patients without HA (matched 1:3) n = 3150 1.7%; *P* < .0001 5.2%; *P* < .0001 0.6%; *P* = .013 3.7%; *P* < .0001 0.3%; *P* < .0001		No
Berger et al. 2016 [22]	HA/B ≥40 y	NR	% (95% CI) Coronary artery/heart disease Ischemic cerebrovascular disease/stroke	Persons with HA/B (H^3^ study) n = NR 60-69 y: 8.1% (3.3-16.1) 70-79 y: 11.8% (5.2-21.9) ≥40 y: 2.5% (1.3-4.2)			General population (DEGS1 study) n = NR 60-69 y: 19.5% (15.9-23.7); *P* = .02 70-79 y: 30.5% (25.9-35.5); *P* = .002 ≥40 y: 3.3% (2.6-4.2); *P* = .35		Yes
Miesbach et al. 2017 [23]	HA/B ≥60 y	2005-2010	Ischemic heart disease	Elderly people with HA/B n/N/% (95% CI) Ages 60-69: 8/107/7.5 (2.4-12.5) Ages 70-79: 9/61/14.8 (5.6-23.9)			Age-matched general population % (95% CI) Ages 60-69: 19.5 (15.9-23.7); *P* = .001 Ages 70-79: 30.5 (25.9-35.5); *P* = .008		Yes
Sood et al. 2018 [24]	HA/B 54-73 y	2012-2015	n, % CVD Myocardial infarction Transient ischemic attack Ischemic or embolic stroke Venous thromboembolism DVT and PE DVT only PE only	HA/B N = 200 30, 15% 15, 7.5% 3 1 6 2 2 2	HA/B with CVD (ARIC definition) 15.0%	HA/B with CVD (NHANES definition) 10.0%	Matched with CVD (ARIC definition) 25.8%	Matched with CVD (NHANES definition) 17.9%	Yes
Humphries et al. 2018 [25]	HA All ages	1995-2014	% Stroke Coronary artery disease Arterial thrombosis Venous thrombosis	Persons with HA n = 74 2.7% 6.8% 0.0% 4.1%			Matched controls n = 222 5.0% 14.9% 0.5% 5.9%		Yes
Lövdahl et al. 2019 [26]	HA/B ≥30 y	NR	% Myocardial ischemia	Persons with hemophilia n = 1431 8.2%			Matched controls n = 7150 8.1%		Comparable
Faghmous et al. 2021 [27]	HA All ages	2000-2019	Myocardial infarction Adjusted IRR (95% CI) Pulmonary embolism Adjusted IRR (95% CI) Ischemic stroke Adjusted IRR (95% CI) Deep vein thrombosis Adjusted IRR (95% CI)	0.80% (95% CI, 0.53-1.12) n = 3494 1.23 (0.82-1.86) 0.29% (95% CI, 0.14-0.49) n = 3491 0.89 (0.45-1.77) 1.03% (95% CI, 0.72-1.39) n = 3494 1.48 (1.01-2.16) 0.89% (95% CI, 0.60-1.23) n = 3482 1.53 (1.00-2.32)			0.54% (95% CI, 0.44-0.66) n = 16,378 0.27% (95% CI, 0.20-0.35) n = 16,382 0.57% (95% CI, 0.46-0.70) n = 16,377 0.47% (95% CI, 0.37-0.58) n = 16,334		Comparable for myocardial infarction and PE Slightly higher for ischemic stroke and DVT in persons with hemophilia

ARIC, atherosclerosis risk in communities; CVD, cardiovascular disease; DVT, deep vein thrombosis; DEGS, German Health Interview and Examination Survey for Adults; DVT, deep vein thrombosis; HA, hemophilia A; HA/B, hemophilia A/B; NHANES, National Health and Nutrition Examination Survey; NR, not reported; PE, pulmonary embolism; IRR, incidence rate ratio; SPR, standardized prevalence ratio.

a Rate = discharges per 1000 population.

b Prevalence in Germany as percentage of total population. Source: Federal Statistical Office Germany.

c SPRs were calculated to compare the prevalence of CVD risk factor in the study cohort (N = 185) with that of US non-Hispanic White males aged ≥18 years (NHANES data from 2005 and 2006).

### 3.4 Mortality

There were 29 studies containing mortality data, with 9 studies reporting standardized mortality ratios (SMRs) comparing mortality in the hemophilia population with that in the general population (Table 3) [2,9,1,[28], [29], [30], [31], [32], [33]]. Across the 9 publications, all-cause SMRs (excluding those split by severity) range from 0.89 to 2.3, indicating that mortality is generally higher in those with hemophilia. Two studies examined changes in mortality between 2 separate time frames for persons with hemophilia in Italy and Brazil [2,32]. These publications showed that historically, mortality was generally higher for hemophilia populations (1990-1999, 1.98 [2]; 2000-2002, 1.51 [32]). However, more recent SMR data show that mortality is in fact comparable with that in the general population (2000-2007, 1.08 [2]; 2012-2014, 0.89 [32]). Mortality due to CVD is consistently lower in persons with hemophilia, with SMRs ranging from 0.25 to 0.62. Death due to ICH is considerably higher in hemophilia population than that in the general population (SMRs range from 3.5 to 14.88). One study by Day et al. [34] not included in Table 3 reported in-hospital mortality for admissions with hemophilia from 2017 and compared against all admissions. Although no numerical data are provided in that study, visual data show that mortality appears to be reduced in persons with hemophilia codiagnosed with nontraumatic ICH, acute myocardial infarction, and stroke when compared to all hospital admissions.

**Table 3:** Mortality in persons with hemophilia vs the general population.

Reference	No. of patients	Type	Time frame	Country	All-cause SMR or HR (95% CI)	Cause-specific SMR (95% CI)	All-cause SMR or HR by hemophilia type (95% CI)	Mortality rate
Plug et al. 2006 [10]	967	HA/B	1992-2001	Netherlands	All All patients: 2.3 (1.9-2.8) HIV negative: 1.7 (1.3-2.1) Severe All patients: 5.1 (3.8-6.8) HIV-negative: 2.8 (1.9-4.2) Moderate All patients: 2.6 (1.5-4.3) HIV negative: 2.3 (1.3-3.9) Mild All patients: 1.3 (0.9-1.9) HIV negative: 1.2 (0.8-1.6)	Ischemic heart disease 0.5 (0.2-1.1) Cerebrovascular disease 1.0 (0.2-2.2)	HA All patients: 2.3 (1.9-2.9) HIV-negative: 1.7 (1.4-2.2) HB All patients: 2.3 (1.3-4.0) HIV-negative: 1.3 (0.6-2.7)	NR
Darby et al. 2007 [9]	6018	HA/B	1977-1998	United Kingdom	1.43 (1.34-1.54)	Intracranial hemorrhage 14.88 (12.40-17.72) Ischemic heart disease 0.62 (0.51-0.76) Ischemic stroke 0.63 (0.17-1.62)	NR	NR
Tagliaferri et al. 2010 [2]	6632	HA/B Severe	1980-2007	Italy	1990-1999: 1.98 (1.54-2.51) 2000-2007: 1.08 (0.83-1.40)	Cardiovascular disease 1990-1999: 0.25 (0.11-0.49) 2000-2007: 0.55 (0.29-0.93)	HA severe 1990-1999: 4.08 (2.65-6.25) 2000-2007: 3.30 (1.83-5.67) HB severe 1990-1999: 7.94 (3.21-19.81) 2000-2007: 2.18 (0.69-7.76)	Crude mortality rate (×1000) (95% CI) 1990-1999: 6.41 (5.63-7.19) 2000-2007: 5.10 (4.15-5.98) Standardized mortality rate (×1000) (95% CI) 1990-1999: 7.03 (6.13-8.01) 2000-2007: 4.83 (3.85-5.84)
Lövdahl et al. 2013 [28]	1431	HA/B	1968-2009	Sweden	HR: 2.2 (1.8-2.7)	NR	HA and HB severe HR: 6.6 (4.5-10.0)	NR
Tu et al. 2013 [29]	988	HA/B	1997-2007	Taiwan	All severities 1.3 (0.9-1.9) Severe 2.1 (1.7-2.7) Moderate 1.4 (1.0-1.9) Mild 0.7 (0.4-1.0)	NR	HA All severities: 1.4 (1.0-1.9) Severe: 2.1 (1.7-2.7) Moderate: 1.4 (1.0-1.9) Mild: 0.8 (0.5-1.1) HB All severities: 1.0 (0.8-1.4) Severe: 2.0 (1.6-2.6) Moderate: 1.0 (0.8-1.4) Mild: 0.3 (0.1-0.6)	Crude mortality rate (95% CI) per 100,000 males Per year (HA+HB) All severities: 574.0 (528.0-622.9) Severe: 639.1 (590.4-690.5) Moderate: 520.1 (476.3-566.7) Mild: 504.3 (460.9-550.0) Age-standardized mortality rate (95% CI) per 100,000 males per year (HA+HB) All severities: 693.3 (642.4-746.6) Severe: 719.0 (667.4-773.5) Moderate: 587.6 (541.4-637.5) Mild: 521.3 (477.2-567.7)
Chang et al. 2014 [30]	NR	HA/B	1997-2009	Taiwan	1.98 (NR)	NR	NR	Standardized crude death rate 10.2 per 1000 people 5.87 per 1000 people (general population in 2009)
Loomans et al. 2017 [31]	2709	HA nonsevere	1980-2010	Europe and Australia	NR	Intracranial hemorrhage 3.5 (2.0-5.8)	NR	NR
Jardim et al. 2019 [32]	784 Deaths	HA/HB	2000-2014	Brazil	Overall: 1.13 (1.01-1.16). 2000-2002: 1.51 (1.29-1.74) 2012-2014: 0.89 (0.74-1.04)	NR	NR	NR
Hassan et al. 2021 [33]	1031	HA/B	2001-2018	Netherlands	1.4 (1.2-1.7)	Ischemic heart disease 0.3 (0.1-0.9) Ischemic stroke 1.1 (0.03-6.0) Intracranial bleeding 12.8 (7.8-19.8)	NR	Crude death rate 8.9 per 1000 person-y. 8.2 per 1000 person-y (general population 2001-2017).

HA/B, hemophilia A/B; HR, hazard ratio; NR, not reported; SMR, standardized mortality ratio.

## 4 Discussion

In this scoping review, a literature search was performed to identify and summarize currently available data on the prevalence of the selected adverse events in hemophilia and general populations. The prevalence of the bleeding events selected for analysis (hemorrhagic stroke, ICH, and gastrointestinal bleeding) was consistently higher in those with hemophilia than in nonhemophilic controls, with serious bleeding events such as ICH showing a high mortality rate. However, although it has been proposed that persons with hemophilia could be protected from thrombosis [5], the prevalence of the selected thrombotic events (myocardial infarction, ischemic stroke, and arterial and/or venous thrombosis) was not always higher in the general population, with a small number of studies reporting a higher prevalence in hemophilia populations. Based on the current literature, it is not possible to conclude that persons with hemophilia are protected from thrombosis or that the prevalence of the selected adverse events is approaching that of the general population.

### 4.1 Comparison of studies identified in the search

As described in the following section, the conclusions of this study align with several published reviews examining bleeding, thrombosis, and mortality in persons with hemophilia [[35], [36], [37], [38], [39]].

#### 4.1.1 Bleeding

ICH remains a severe bleeding event for persons with hemophilia that results in high mortality. ICH is considered to occur more frequently in 2 age groups: children ≤2 years and adults ≥60 years with associated risk factors such as hypertension [40]. In our searches, 2 publications reported ICH prevalence in newborns with hemophilia (3.1%-3.4%), which was higher compared to the general population (0.04%-0.11%) [14,16]. As newborns with hemophilia are at a high risk of ICH, current guidelines recommend planned cesarean section for delivery of the affected or potentially affected infants [41]. Two publications reported higher prevalence of nonfatal ICH in adults with hemophilia aged ≥30 years (1.6% vs 0.4%) [4] or ≥40 years (7.4% vs 0.4%) [5]. In both studies, approximately one-fourth of the adult patients were receiving prophylactic treatment (23% and 27%, respectively), highlighting the importance of life-long prophylaxis to prevent serious bleeding events as well as the need to optimize prophylaxis regimens to ensure best coverage.

A recent systematic review and meta-analysis provides an in-depth analysis of the incidence and mortality of ICH in persons with hemophilia [39]. As described in the current study, incidence rates of ICH were found to be higher compared to the general population across all age groups, and the ICH risk was especially high in hemophilic neonates. Although neonates are especially vulnerable, it is important to monitor patients of all ages. Underlying risk factors such as hypertension, which has a higher prevalence in hemophilia vs the general population [42], should be addressed in accordance with current treatment guidelines [43] to mitigate the occurrence of ICH.

#### 4.1.2 Thrombosis

Interestingly, although several studies report that the prevalence of thrombotic events is higher in the general population, a small number of studies appear to show that the prevalence of some thrombotic events is comparable or in fact higher in those with hemophilia [[6], [7], [8],^27^]. Three publications from Pocoski et al. [7] and Humphries et al. [6] were identified in the search, with 2 reporting a higher prevalence of thrombotic events in the general population and the third instead reporting a higher prevalence in those with hemophilia [25]. The first study, which was published in 2014, showed that the prevalence of cardiovascular comorbidities was found increased in persons with hemophilia A included in the MarketScan Commercial and Medicare Research Databases [7]. In 2016, these findings were confirmed in a second patient population from the US [6]. However, a third publication in 2018 that used medical records from the Henry Ford Health System was unable to replicate these findings [25]. As alluded to in the latter publication, differences in databases used between these 3 studies and the fact that at least 2 medical visits were required in the third study meant that controls could have had a higher medical burden and subsequently also have had higher rates of CVD [25]. The third study also had a comparably low sample size of persons with hemophilia (n = 74). Work from other groups has also reported higher or comparable levels of thrombotic events when comparing hemophilia and general populations [8,26,27].

Based on the data identified, the prevalence of arterial thrombotic events in persons with hemophilia (myocardial infarction and ischemic stroke) appears to be greater when compared with that of venous thrombotic events (deep vein thrombosis and pulmonary embolism). However, this was not observed in the general population. Patients with hemophilia had a slightly reduced prevalence of venous thrombosis than the general population, which is in agreement with the data from a previous study that suggested that persons with hemophilia may potentially exhibit some protection from venous thrombosis, but not arterial thrombosis [44]. However, the paucity of data makes these comparisons difficult, and more research is needed to understand these differences. As discussed previously in the literature, cardiovascular risk factors such as atherosclerosis that contribute to arterial thrombosis are found equally in persons with hemophilia and general population [3]. It is therefore increasingly important that the underlying cardiovascular risk factors are investigated and addressed in persons with hemophilia as they are treated with replacement products and encounter new and improved hemostatic therapies. Current treatment guidelines state that persons with hemophilia should receive the same screening and management of cardiovascular risk factors as the general population [43].

Previous reviews have also addressed this topic. Rizwan et al. [37] published a scoping review examining the prevalence of CVD in general and cardiovascular risk factors in persons with hemophilia based on 30 original articles and reviews published between 1983 and 2012. There was also conflicting evidence supporting the possibility that hemophilia exhibits a protective effect against CVD, which aligns with the overall conclusion of the current study. A recent narrative review also summarizes the prevalence of cardiovascular risk factors in persons with hemophilia and promotes increased awareness of CVD in an aging hemophilia population [38]. Prospective studies to understand whether persons with hemophilia are indeed at a lower risk of thrombotic complications and whether this is changing over time are lacking. A recent publication by Van der Valk et al. [45] described the results from a prospective, multicenter, observational study evaluating the incidence of CVD in persons with hemophilia from the Netherlands and United Kingdom over a 5-year period. The QRISK2-2011 CVD risk tool was used to determine the predicted CVD event rate in the general population for comparison. CVD incidence in persons with hemophilia was found to be lower than that predicted by QRISK2-2011 (relative risk [95% CI], 0.38 [0.18-0.80]), which supported the idea that persons with hemophilia are protected against CVD. Longer-term prospective studies will be informative in the future to understand this more given the changing treatment landscape.

#### 4.1.3 Mortality

Improvements in hemophilia treatment and patient care have resulted in increased life expectancy for persons with hemophilia over recent decades. Specific studies by Tagliaferri et al. [2] and Jardim et al. [32] have reported reductions in mortality over time in persons with hemophilia from Italy and Brazil. An increasingly aging population of persons with hemophilia could translate to an increasing number of patients expected to develop CVD in the future, mirroring patterns in the general population. As reported by Tagliaferri et al. [2], the cause-specific SMR for CVD has increased from 0.25 (1990-1999) to 0.55 (2000-2007).

Overall, a comparison of SMRs between different studies and patient populations is challenging, and these data should be interpreted with caution. The comparison of SMRs provides a less biased approach than the comparison of crude mortality rates between studies. Comparisons of SMRs across countries may not always be appropriate as different countries may have access to different treatments and patient care standards may vary. To make more accurate comparisons across countries, SMRs could be calculated using international reference populations. Regarding cause-specific mortality data, it is sometimes unclear how observed events contributed to death, as data are interpreted differently in different studies. Data was also not reported differently for patients with vs without inhibitors.

Two recent systematic reviews have examined mortality and causes of death in persons with hemophilia [35,36]. Hay et al. [36] concluded that persons with hemophilia A have a higher mortality rate than the general population, but incomplete reporting of data limits evidence on mortality. The systematic review and meta-analysis published by Alem et al. [35] provided an overall SMR of 1.93 calculated from data cross 9 studies, indicating that persons with hemophilia are at a greater risk of death than matched controls. Analysis of SMR data from studies published before (SMR: 2.40) and after 2000 (SMR: 1.20) also supports the notion that mortality is decreasing over time due to the changes in the treatment landscape.

### 4.2 Implications for future clinical practice and research

Non–factor-replacement therapies are now either available (emicizumab) or are under clinical trials (concizumab, marstacimab, and fitusiran) as novel therapeutics for persons with hemophilia. One major advantage associated with these treatments relative to factor replacement therapy is the improvement in the quality of life due to subcutaneous administration, flexible dosing, and consistent levels with the avoidance of peaks and troughs. There has been a low occurrence of treatment-emergent reactions and a low incidence of neutralizing/nonneutralizing antibodies observed with the use of non–factor-replacement therapies [[46], [47], [48], [49], [50], [51], [52], [53], [54], [55]]. Nonreplacement therapies have been associated with some thrombotic effects in clinical trials, often with the concomitant use of other hemostatic agents or other specific thromboembolic risk factors [47,56,57]. Relevant amendments were made to trial protocols for emicizumab [47], concizumab [57], and fitusiran [58] to mitigate these risks by providing more detailed guidance on the management of breakthrough bleeds. Further research and data are needed to elucidate these clinical and nonclinical risks and benefits associated with these therapies.

### 4.3 Strengths and limitations

Over 900 published abstracts were screened to identify relevant prevalence and mortality data of persons with hemophilia and the general population. This analysis therefore provides a comprehensive overview of the currently available prevalence data of several individual adverse events in persons with hemophilia vs nonhemophilic controls. However, direct comparison of data between the studies identified is limited due to the variation in the data sources used, the sizes and geographic distributions of the studied patient populations, and statistical methods used for analysis. For example, prevalence data were often derived from large patient databases from different sources, including medical records [4,5,8,20,21,[23], [24], [25], [26]] or commercial/insurance databases [6,7,15,19]. Although some studies searched records using predefined criteria for hemophilia and comorbidities [4,5,8,[21], [22], [23], [24]], others used International Classification of Diseases codes to select individuals who had experienced adverse events [6,7,15,19,20,25,26]. These factors, therefore, greatly limited the ability to accurately compare studies to evaluate if persons with hemophilia are protected against thrombosis and whether the prevalence of cardiovascular events in persons with hemophilia is increasing over time.

## 5 Conclusion

Cerebral bleeding is a serious bleeding event in persons with hemophilia, which shows a high mortality rate. Persons with hemophilia are affected by both arterial and venous thrombotic events. Although it is unclear whether persons with hemophilia are protected from arterial thrombosis, there may potentially be some protection from venous thrombosis. However, this observation needs to be followed up with novel treatments to understand more. These conclusions might be impacted by limitations in the type and size of the studies analyzed. Further prospective studies are required to understand the risk of thrombotic complications in persons with hemophilia and whether this is changing over time with a changing treatment landscape.
