# Supplementary material for: Prevalence of selected bleeding and thrombotic events in persons with hemophilia versus the general population: A scoping review
Source: Res Pract Thromb Haemost. 2022 Dec 9;7(1):100007. doi: 10.1016/j.rpth.2022.100007 (PMC9986102; doi:10.1016/j.rpth.2022.100007)
Supplement: Supplementary Table S1 [file mmc1.docx]

**Supplementary Table 1. Search Strategy**

| **Set#** | **Searched for** |
| --- | --- |
| S1 | EMB.EXACT("brain hemorrhage") OR EMB.EXACT.EXPLODE("upper gastrointestinal bleeding") OR EMB.EXACT("chronic hepatitis C") OR MESH.EXACT("Intracranial Thrombosis") OR MESH.EXACT("Intracranial Arterial Diseases") OR MESH.EXACT("Intracranial Hemorrhage, Traumatic") OR MESH.EXACT("Intracranial Embolism") OR MESH.EXACT("Intracranial Arteriovenous Malformations") OR MESH.EXACT("Intracranial Aneurysm") OR MESH.EXACT("Intracranial Hemorrhage, Hypertensive") OR MESH.EXACT("Intracranial Embolism and Thrombosis") OR MESH.EXACT("Intracranial Arteriosclerosis") OR MESH.EXACT("Intracranial Hemorrhages") OR MESH.EXACT("Gastrointestinal Hemorrhage") OR MESH.EXACT("Hepatitis C, Chronic") OR MESH.EXACT("Hepatitis C") or emb.exact.explode(mortality) or mesh.exact(mortality) or emb.exact.explode(death) or mesh.exact(death) |
| S2 | ti,ab((intracranial or cranial) near/3 (haemorrhage* or hemorrhage* or bleed*)) |
| S3 | ti,ab((gastric* or gastro* OR stomach or intestine* or Gastrointestinal) near/5 bleed*) OR ti,ab((traum* or surgical or surgery or spontaneous*) near/3 bleed*) |
| S4 | ti,ab("Hepatitis C") |
| S5 | MESH.EXACT("Thrombotic Microangiopathies") OR MESH.EXACT.EXPLODE("Embolism and Thrombosis") |
| S6 | EMB.EXACT.EXPLODE("thromboembolism") OR EMB.EXACT.EXPLODE("thrombosis") |
| S7 | TI,AB(arterial thrombo* OR Deep vein blood clot* OR deep vein thrombophlebitis OR deep vein thromb* OR DVT OR disseminated intravascular coagulation OR Femoral Vein Thromb* OR mesenteric ischaemia OR mesenteric ischemia OR Myocardial Infarction OR Portal Vein Thrombo* OR pulmonary embol* OR Thromboembolic OR Thrombophlebitis OR thrombotic event* OR thrombotic microangiopath* OR thrombotic stroke* OR thrombocytopenic purpura OR venous thromboembolism OR venous thrombosis OR myocardial or infarction or infarct or stroke or STEMI or mortality or death* or died) |
| S8 | TI,AB(THROMBUS OR THROMBOSIS) |
| S9 | S8 OR S7 OR S6 OR S5 OR S4 OR S3 OR S2 OR S1 |
| S10 | ti,ab(haemophilia OR hemophilia OR haemophiliac OR hemophiliac or "inherited bleeding disorder*" or "rare bleeding disorder*" or hemophilic or haemophilic or "congenital bleeding disorder*") |
| S11 | ti(PWH or PWHA or PWHB or inhibitor* or PWHI or PWHAI) AND ((ti,ab(haemophilia OR hemophilia OR haemophiliac OR hemophiliac)) OR EMB.EXACT("hemophilia B") OR EMB.EXACT("hemophilia A") OR EMB.EXACT("hemophilia") OR MESH.EXACT("Hemophilia B") OR MESH.EXACT("Hemophilia A")) |
| S12 | EMB.EXACT("hemophilia B") OR EMB.EXACT("hemophilia A") OR EMB.EXACT("hemophilia") |
| S13 | MESH.EXACT("Hemophilia B") OR MESH.EXACT("Hemophilia A") |
| S14 | S13 OR S12 OR S11 OR S10 |
| S15 | S14 AND S9 |
| S16 | S15 not TI,AB("ANIMAL" OR ANIMALS OR CAT OR CATS OR DOG OR DOGS OR FISH OR RABBIT OR RABBITS OR RAT OR RATS OR COW OR COWS OR CAMELS OR CAMEL OR MOUSE OR MICE OR MURIDAE OR RODENT OR RODENTAE OR PRIMATE* OR MONKEY* OR CHIMP* or horse or horses or "in vitro" or "in vivo") |
| S17 | S16 not (rtype.exact("English Abstract" OR "Patent" OR "Conference Abstract" OR "Case Reports" OR "Meeting Abstract" OR "Conference Paper" OR "Meeting Poster" OR "Editorial" OR "Conference Review" OR "Guideline" OR "Meeting Summary" OR "Biography" OR "Congress" OR "Clinical Conference" OR "Legal Case" OR "Portrait" OR "Bibliography" OR "Interview" OR "Lecture" OR "Meeting Paper" OR "Meeting Report" OR "Personal Narrative" OR "Technical Report")) |
| S18 | (S17 not (emb("case report" or "case study" or "case series") or mesh("case report" or "case study" or "case series"))) and (pd(20050101-20201231)) |
| S19 | S18 NOT ti("in a patient") |
| S20 | S19 not ti(acquired hem* or acquired haem*) |
| S21 | S20 and la(english) |
| S22 | S21 not ti(leukaemia* or leukemia OR lymphoma OR myeloproliferative OR myeloma or rituximab) |
| S23 | S14 and mortality and (malignan* or cancer*) |
| S24 | S14 AND ((BLEED OR BLEEDS OR BLEEDING) NEAR/2 EVENT*) |
| S25 | S22 AND TI,AB(HEMOTHORAX OR HEMATURIA OR Hemoperitoneum) |
| S26 | (S25 OR S24 OR S23) and (pd(20050101-20211231) NOT rtype.exact("Case Reports" OR "Editorial" OR "English Abstract" OR "Note" OR "Comment" OR "Conference Review" OR "Meeting Summary")) |
| S27 | S26 OR S22 |

* Duplicates were removed from the search, but included in the result count.
